# Oral Microbiota Features in Subjects with Down Syndrome and Periodontal Diseases: A Systematic Review

**DOI:** 10.3390/ijms22179251

**Published:** 2021-08-26

**Authors:** Maria Contaldo, Alberta Lucchese, Antonio Romano, Fedora Della Vella, Dario Di Stasio, Rosario Serpico, Massimo Petruzzi

**Affiliations:** 1 Multidisciplinary Department of Medical-Surgical and Dental Specialties, University of Campania Luigi Vanvitelli, Via Luigi de Crecchio, 6, 80138 Naples, Italy; alberta.lucchese@unicampania.it (A.L.); antonio.romano4@unicampania.it (A.R.); dario.distasio@unicampania.it (D.D.S.); rosario.serpico@unicampania.it (R.S.); 2 Interdisciplinary Department of Medicine, University of Bari “Aldo Moro”, 70121 Bari, Italy; dellavellaf@gmail.com (F.D.V.); massimo.petruzzi@uniba.it (M.P.)

**Keywords:** Down syndrome, trisomy 21, microbiome, microbiota, periodontitis, gingivitis, periodontal diseases, bacteria, fungi, virus, dental biofilm, dental plaque

## Abstract

Down syndrome (DS) is a genetic disorder associated with early-onset periodontitis and other periodontal diseases (PDs). The present work aimed to systematically review the scientific literature reporting studies in vivo on oral microbiota features in subjects with DS and related periodontal health and to highlight any correlation and difference with subjects not affected by DS, with and without PDs. PubMed, Web of Science, Scopus and Cochrane were searched for relevant studies in May 2021. The participants were subjects affected by Down syndrome (DS) with and without periodontal diseases; the study compared subjects with periodontal diseases but not affected by DS, and DS without periodontal diseases; the outcomes were the differences in oral microbiota/periodontopathogen bacterial composition among subjects considered; the study design was a systematic review. Study quality was assessed with risk of bias in non-randomized studies of interventions (ROBINS-I). Of the 954 references retrieved, 26 studies were considered. The conclusions from the qualitative assessment of the papers revealed an increasing knowledge over the last years of the microbiota associated with DS and their periodontal diseases, in comparison with healthy subjects and subjects with other kinds of mental disabilities. Few data have emerged on the mycobiome and virobiome of DS, hence, further investigations are still necessary.

## 1. Introduction

### 1.1. Down Syndrome

Down syndrome (DS) is an autosomal genetic disorder resulting from an extra 21st chromosome (trisomy 21) [1]. DS is the most common human aneuploidy compatible with life and its estimated prevalence is between 1 per 800–1100 live births worldwide, and is associated with advanced maternal age [2,3].

Persons with DS are characterized by generalized growth deficiencies, mild to severe cognitive development, congenital cardiac defects, and are predisposed to suffer leukemia hypertension, gastrointestinal problems and early onset of Alzheimer’s disease [4]. DS subjects also present a characteristic phenotype. The most suggestive dermatological manifestations include *alopecia areata*, palmoplantar hyperkeratosis and premature hair greying and ageing [5]. The typical facial features are as brachycephaly, flat nasal bridge, epicanthic fold, narrowed and slanted eye slits, cataracts, defects of vision and strabismus. The oral district is characterized by dental anomalies (numerical, developmental and eruptive), geographic tongue, fissured tongue, lip fissures and cheilitis, recurrent oral candidiasis [6,7,8], microstomia, macroglossia, advanced tongue position and small chin, resulting in the classical phenotype of a protruding tongue with lip incompetence [1].

Trisomy 21 is also associated with defective neutrophil and monocyte chemotaxis and phagocytosis [9,10], plus T system immune-deficiencies [11] and premature immune system senescence [12]. These conditions predispose DS subjects to chronic proinflammatory status and increase in oxidative stress due to mitochondrial dysfunction which, concomitant to metabolic diseases such as diabetes [13], are also responsible for higher susceptibility to oral and extra-oral infection, than in euploid and immunocompetent subjects.

Furthermore, DS subjects are more predisposed to early-onset and rapidly progressive periodontal diseases, such as gingivitis, localized and generalized juvenile periodontitis and chronic periodontitis [14], further aggravated by their motor disabilities limiting their performance in oral hygiene practices [15].

### 1.2. Periodontal Diseases

Periodontal diseases (PDs) include a series of inflammatory diseases affecting the supporting structures of the teeth (gingiva, periodontal ligaments, alveolar bone and dental cementum) and are sixth among the most prevalent human diseases [16], thus being considered a global disease burden [17] and one of the most prevalent forms of bone destructive pathology in humans [18,19].

In 2018, the classification of PDs has been revised and updated, with the accordance of several international scientific societies and experts [20,21]. According to the novel classification of PDs, it is possible to identify and distinguish a series of periodontal diseases and conditions, grouped as follows:

- Periodontal health, gingival diseases and conditions: healthy periodontium and gingiva; gingivitis dental biofilm-induced; gingival diseases non-dental biofilm-induced.
- Periodontitis: necrotizing periodontal diseases; periodontitis; periodontitis as a manifestation of systemic disease.
- Other conditions affecting the periodontium: systemic diseases or conditions affecting the periodontal support tissues; periodontal abscesses and endodontic-periodontal lesions; mucogingival deformities and conditions; traumatic occlusal forces; tooth and prosthesis related factors.

Based on this classification, periodontal diseases occurring in DS are ascribed in the second group, as “periodontitis as a manifestation of systemic disease” [20], given that the systemic condition favors and worsens the onset and the course of periodontitis, which, in DS, has an early onset of severity higher than in non-DS subjects.

In the present work, the term “PDs” is used to generally refer to gingivitis and periodontitis, two plaque-related inflammatory diseases of the gingiva and periodontal structures, respectively.

Gingivitis is an acute or chronic inflammation of the superficial gingiva, diffuse or localized, characterized by bleeding—spontaneous or on probing, reddened and plump gums, which affects subjects with scarce or bad oral hygiene, which tend to accumulate mature plaque around the teeth, thus acting as an inflammatory stimulus for the onset of gingivitis. Periodontitis is, instead, a chronic and progressive disease, triggered by specific periodontopathogen bacteria accumulating supra- and sub-gingivally, in the gingival sulcus, which promote chronic inflammation in predisposed subjects [22,23], responsible for alveolar bone destruction, loss of periodontal attachment and dental mobility, up to tooth loss in severe and untreated cases, at the age of 50 or more [24,25,26]. The course of periodontitis is progressive, and its onset is associated with genetic and environmental factors [27,28,29]. The trigger event which starts the periodontitis in predisposed subjects is the persistence of bacterial plaque and biofilm at the gingival sulcus, when colonized by periodontopathogen bacterial species. The main species involved in the process are a series of Gram-negative/facultative anaerobic bacteria [28] that, accumulating and organizing in a periodontopathogen biofilm in the gingival sulcus, favor the host’s inflammatory over-reaction which perpetuates the local inflammation and periodontal destruction [30,31].

Aside from periodontal implications, periodontopathogen plaque biofilm influence and is influenced also by a series of systemic diseases and conditions [32], proved to be associated with the qualitative and quantitative changes occurring in the oral microbiota during periodontitis, and responsible for the onset of oral infections, as well as triggering and/or worsening of a series of systemic diseases [33,34,35,36,37,38,39,40,41,42].

### 1.3. Composition of Supragingival and Subgingival Plaque

Dental plaque is a dynamic entity made up of salivary proteins, minerals and, mainly, bacteria, which mutually benefit from the coaggregation, adhesion and metabolic interactions [43] and arrange in a biofilm on the hard and soft tissue of the mouth. The polymicrobial biofilm, which composes dental plaque changes over time and maturates according to diet, oral hygiene habits and location. In general, early and mature plaques, supragingival and subgingival plaques strongly differ from each other in bacterial composition.

Supragingival plaque, considered the main cause of caries and demineralization of enamel ad dentin, is prevalently made up by cariogenic bacteria, such as *Streptococcus mutans*, *Lactobacilli* and *Actinomycetes* species (spp.) as second colonizers, but it also plays a key role in the late coaggregation of periodontopathogen bacteria in the subgingival plaque, consisting mainly of Gram-negative anaerobic bacteria strongly associated with periodontal diseases [44].

**Figure 1:**
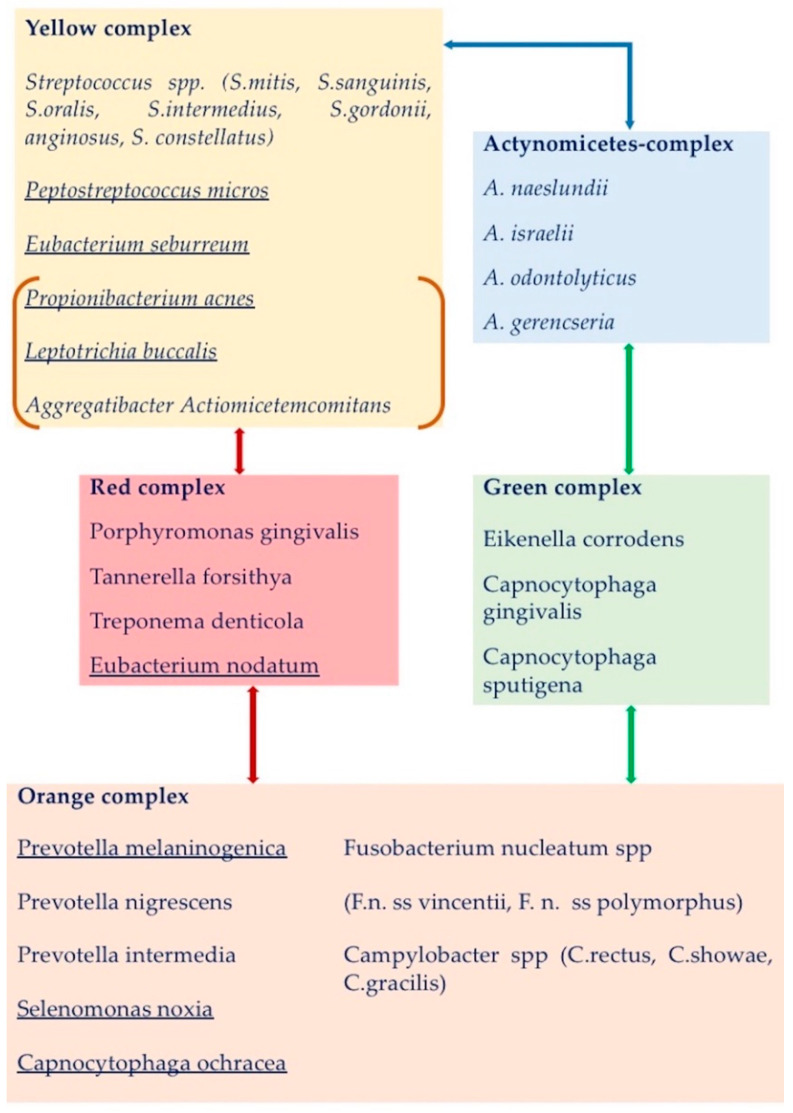
The periodontopathogen potential of each bacterial complex of the supragingival mature plaque and their relationships, according to Haffajee et al., 2008 [46]. The bacteria found in clusters different from the ones of the subgingival plaque are underlined. Actinomyces complex (in blue box) and the yellow complex were tightly associated. The red complex bacteria were associated with both the yellow and orange complexes. The green complex was interrelated with the Actinomyces spp. and the orange complex species. The bacteria in orange square brackets (*A. actinomycetemcomitans*, *P. acnes*, and *L. buccalis*) were occasionally found associated in the yellow complex. *Neisseria mucosa* and *Veillonella parvula* of the purple complex were not associated with other complexes, while *Treponema socranskii* and *Gemellia morbillorum* related to different clusters and are not shown.

The works by Haffajee, Socransky et al. first [45,46] and the study on oral microbiome composition recently [47,48,49], have elucidated the bacterial composition of supra-and subgingival bacteria in the dental plaque, thanks to the use of culture-independent high throughput approach technologies, such as reverse transcriptase-polymerase chain reaction (RT-PCR) [50], checkerboard DNA–DNA hybridization (CDDH) [45], and next generation sequencing [51], which allow the simultaneous evaluation of large numbers of microbial species from different samples—saliva, supra- and sub-gingival plaque—and from a wide range of subjects. To date, the oral microbiota results composed of more than 600 microbial species—eukaryotes, archaea, bacteria, fungi and viruses—living in specific ecological niches of the mouth [47,48], 169 of which constitute the indigenous “core oral microbiome”, and are qualitatively and quantitatively housed in three different intraoral niches, classified as follows: Group 1, buccal mucosa, keratinized gingiva and hard palate; Group 2, saliva, tongue, tonsils and throat (back wall of oropharynx); Group 3, sub- and supra-gingival plaque [49]. Further details about subgingival and supragingival periodontopathogen bacteria, classified and color-labeled according to their periodontopathogen potential by Socransky et al. and Haffajee et al. [45,46] have been reported in Table 1 and Figure 1.

### 1.4. Periodontal Diseases in Down Syndrome

As reported previously, based on the novel classification of periodontal diseases, those occurring in DS are a manifestation of systemic disease, being DS associated with defective host-response [52] and increased pro-inflammatory cytokines productions, which favor, anticipate and worsen the onset and the course of periodontitis in these subjects. In detail, Scalioni et al. reported in a recent systematic review the scientific evidence of an association between PDs and DS [53]. The authors reported worst and higher periodontal parameters among patients with DS than among those without DS, also in similar oral hygiene and plaque conditions, suggesting that the aggressiveness and the early onset of periodontitis, as well as the higher prevalence of gingivitis in DS may be related to their systemic compromised immunological conditions, which favor the early subgingival colonization and growth of periodontopathogen bacteria.

On these bases, the present review aimed to establish and identify any possible difference in the oral microbiota of DS compared to healthy non-DS subjects, which could be responsible for the peculiarities occurring in PDs in DS.

### 1.5. Aim

The present work aimed to systematically review the scientific literature reporting studies in vivo on oral microbiota features in subjects with DS and related periodontal health, to highlight any correlation and difference with subjects not affected by DS, with and without PDs.

## 2. Materials and Methods

The present work was performed according to the Preferred Reporting Items for Systematic Reviews and Meta-Analysis (PRISMA) checklist [54]. The protocol was registered on PROSPERO (reference CRD42021261843) and the research query was formulated according to the PICOS method (Participants, Intervention, Comparison, Outcomes and Study): the participants were subjects affected by Down syndrome (DS) with and without periodontal diseases; the intervention was the comparison with subjects with periodontal diseases but not affected by DS, and DS without periodontal diseases; the outcomes were the differences in oral microbiota/periodontopathogen bacterial composition among subjects considered; the study design was a systematic review.

### 2.1. Research Protocol

The research was conducted on 28 May 2021, and the MeSHs (medical subject headings) terms used and combined by Boolean connectors are reported in Table 2.

### 2.2. Eligibility Criteria

The types of studies eligible for the present research were all the original papers reporting randomized controlled clinical trials and all the observational studies (cohort and case-control studies, cross-sectional investigations, case series and single case reports) describing features related to the oral microbiota/oral microbiome composition in subjects with DS, with and without periodontal diseases. Letters, proceedings, editorials, reviews, theses, abstracts and all studies written in a language other than English were not taken into consideration.

### 2.3. Literature Search, Selection of Studies and Data Extraction

PubMed, Web of Science (WoS), Scopus and Cochrane Library were investigated with no limits of the year of publishing, from the first available date until May 2021. The results of the four searches were imported and combined with Mendeley-Reference Management Software and Researcher Network (available at https://www.mendeley.com (accessed on 21 July 2021). First, the duplicates were removed; then, two reviewers examined the titles and abstracts from the documents and excluded any papers that did not meet the inclusion criteria. For abstracts not providing sufficient information to evaluate the eligibility of the paper, its full text was considered. Later, the full texts of the remaining articles were thoroughly read by two reviewers, to report the relevance of the content of each study and consider those suitable for qualitative synthesis. In any case of disagreement between the two reviewers, a third author was consulted.

At the end of selection and reading, the following data had been extracted from each included study: type of study, the aim of the study, sample size, age-range and sex of the subjects, the periodontal status of the enrolled subjects, the sample of the origin for microbial analysis, methods of microbial identification, micro-organisms investigated, and if the study had considered any correlations between periodontal status and microbial composition.

### 2.4. Risk of Bias (RoB) in Individual Studies

The risk of bias (RoB) for each eligible study of the final selection was independently evaluated by two authors, according with the seven domains-based assessment described by Sterne et al. in the risk of bias in non-randomized studies of interventions (ROBINS-I) [55]: bias due to confounding, selection of participants, classification of interventions, deviations from intended interventions, missing data, measurement of outcomes and selection of the reported results.

According to the ROBINS-I tool, each study was overall classified as follows: “low risk of bias” (LR), when the study met all criteria and was comparable to a well-performed randomized trial; “moderate risk of bias” (MR), if the study appeared to be a non-randomized study but could be considered comparable to a well-performed randomized trial; “serious risk of bias” (SR), when one or more criteria have not been met and one or more plausible biases have severely undermined confidence in the results; “critical risk of bias” (CR) for studies too problematic and reporting a critical bias; “unclear risk of bias” (UR) if one or more criteria were rated as unclear and/or a plausible bias raised some doubts about the results.

## 3. Results

### 3.1. Study Selection

**Figure 2:**
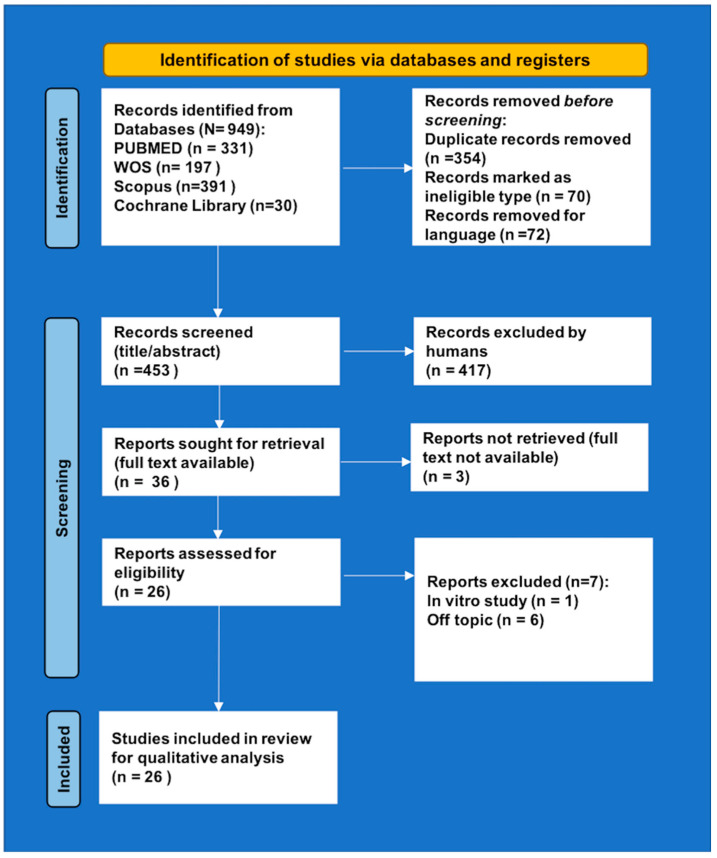
PRISMA 2020 flow diagram.

A total of 949 articles were identified (Figure 2). After removing 354 duplicates and 142 articles as ineligible and/or for language, the remaining 453 records were screened for inclusion/exclusion criteria and title/abstract. After screening for relevance, 417 articles were excluded, and the remaining 36 papers were sought for retrieval. Among them, three full texts were not available (published before the 1970s) and seven were excluded for other reasons. At the end of the selection, 26 original papers [15,51,52,56,57,58,59,60,61,62,63,64,65,66,67,68,69,70,71,72,73,74,75,76,77,78] fulfilled all the requirements and, hence, were considered for qualitative analysis.

### 3.2. Characteristics of the Studies

Among the twenty-six studies considered (all observational studies) published between 1968 and 2021, four were performed in the U.S.A. [56,58,65,72] and in Japan [59,63,66,69], three in Spain [15,51,52], Brazil [64,76,77] and in India [61,74,78], two in Sweden [57,67] and in Greece [68,70], and the remaining came from Germany [60], the Netherlands [67], Saudi Arabia [71], Mexico [73] and Iran [75]. Most observational studies were cross-sectional case/control studies comparing periodontal and microbial features of DS—of various ages—with systematically healthy controls (HC), and/or subjects affected by mental retardations (MR) or cerebral palsy (CP).

With regards to age stages of DS subjects, half of the works focused on young adults, seven on children and adolescents, while the remaining works considered a wider range of ages, although the elderly with DS were rarely investigated.

The bacterial detection in the works written up to 1999 was based on culture approaches, and ELISA (enzyme-linked immunosorbent assay). Later, parallel to the technological improvements and with the arising knowledge on the microbiome, the works from 2000 to today were mainly performed by using molecular and culture-independent high throughput approaches technologies. PCR (polymerase chain reaction) and its variants were the most used, followed by DNA probes, LAMP (loop-mediated isothermal amplification), FISH (fluorescence in situ hybridization), and, just recently, NGS (next generation sequencing).

The periodontal indices adopted to grade the severity of periodontal conditions such as BL (alveolar bone loss), BOP (bleeding on probing), CAL (clinical attachment level), DoP (depth on probing), GBI (gingival bleeding index), GI (gingival index), OHI (oral hygiene index—plaque score), PBS (papillary bleeding score), and PI, plaque index varied according to the age of the work and the classifications available at the age of publication [79,80,81,82,83]. All papers reported at least a descriptive statistical analysis; quantitative and high-levels statistics were mainly reported by the more recent works. Full details about the studies considered have been reported in Table 3. 

The risk of bias (RoB) assessment for each study has been summarized in Table 4.

### 3.3. Outcome

The research criteria without year limitations allowed us to find that the first study related to the association of bacteria and PDs in DS was the American work by Meskin et al. [56], published in 1968. The authors investigated the prevalence of *Bacteroides melaninogenicus* and PDs in a group of 41 DS children with gingivitis and alveolar bone loss, compared with 19 children with cerebral palsy (CP) and general gingival inflammation without bone loss, and 20 additional healthy children (HC) with no gingival inflammation or bone loss. The authors found that *B.* *melaninogenicus* was significantly more prevalent in DS (71%) than in HC (10%), and that, in DS, its prevalence arose according to age, being the lowest (63%) up to 8 years of age, and the highest (86%) at the age of 11–12. Similar percentages (63%) of prevalence were found in the paired group of CP children aged between 7–8 years. Despite these findings, this study has a series of biases that limit its value: first, the heterogeneous sample sizes; second, the difference in the general conditions of the subjects enrolled (DS, CP and not well specified healthy “children from a private pedodontic’s office”); third, the different periodontal status of the subjects in each group (gingivitis and alveolar bone loss in DS, general gingival inflammation and no bone loss in CP; no gingival inflammation or bone loss in HC). Hence it is not clear, from the study, if the different prevalence of *B.* *melaninogenicus* was due to the general (DS) or local (periodontal status) conditions.

In 1992 Bair-Agolme et al. [57] measured the levels of *Actinobacillus actinomycetemcomitans*, Capnocytophaga and Porphyromonas gingivalis in a case-control study on 37 DS, sex- and age-paired with 37 healthy persons (9–21 years). The authors reported a significantly higher prevalence of *A. actinomycetemcomitans* and *Capnocytophaga* in the DS group; these findings did not statistically correlate with the periodontal status of the subjects.

Some works measured indirectly the prevalence of some periodontopathogen bacteria in DS subjects by studying their serum antibodies, as the works by Santos et al. [58], and Morinushi et al. [59]. In 1996, Santos et al. [58] studied the serum antibody titers to *A. actinomycetemcomitans* in sixteen DS (eleven with periodontitis and five with gingivitis) and ten HC without periodontal diseases. They reported a significantly higher presence of Aa-antibodies in the DS groups compared with the healthy controls and the highest titer was found in the group of P-DS. However, from the study design, it was not clear if the low titer of Aa–Ab in the control group were due to the lack of PD or DS. Similarly, Morinushi et al. [59] investigated the relationship between gingivitis and the host response to oral micro-organisms in DS by chronological and dental age, through the measure of the serum antibodies to a series of periodontopathogen bacteria (*Porphyromonas gingivalis*, *Prevotella intermedia*, *Treponema denticola*, *Fusobacterium nucleatum*, *Selenomonas sputigena*, *A. actinomycetemcomitans*, and *Streptococcus mitis*) and compared with 300 HC children. In detail, the authors grouped the children according to Hellman’s dental ages [84], for better defining the relationship between tooth eruption and microbial/immunological measurements, as well due to the delay in eruption present in DS compared to non-DS children. According to Hellman’s classification, Group 1 included children with primary dentition; Group 2 subjects in the eruptive phase of permanent first molars and incisors; Group 3 those in mixed dentition who have completed at least the eruption of permanent first molars and incisors. In children under the age of 4 years, authors reported a significantly higher prevalence of gingival inflammation in DS than in HC (100% vs. 79%). Furthermore, gingival inflammation increased significantly with chronological age (all DS children). With regards to serum antibodies titers, G1—DS reported mean values of the antibody titer to *A. actinomycetemcomitans*, *S. mitis* and *F. nucleatum* higher than those in normal adults, and G3—DS reported significantly higher levels of antibodies to *P. gingivalis* and *A. actinomycetemcomitans*, compared with G1—DS which increased also with chronological ages. A positive correlation was also reported between IgG antibodies to *Porphyromonas gingivalis*, *S. sputigena* and *A. actinomycetemcomitans*.

In 1998, Cichon et al. [60] assessed the periodontal and microbiological status in a group of 10 DS (aged between 20–31) and 11 CP (23–53 years). They did not report any significant differences in the presence and prevalence of a series of periodontopathogen bacteria in the two groups (*A. actinomycetemcomitans*, *P. gingivalis*, *P. intermedia*, *T. denticola*, *F. nucleatum*, *Eikenella corrodens* (*Ec*), *Tennerella forsythia*, *Campylobacter rectus*). In DS, *P. intermedia* was the most frequently identified species (in 50% of subjects), followed by *F. nucleatum* (30%), *T. denticola* and *E. corrodens* (20%), *C. rectus*, *P. gigivalis*, *T. forsythia* (10%). No *A. actinomycetemcomitans* were found in any subject.

In 1998, Sreedevi et al. [61] correlated gingival and periodontal health status and the presence of *A. actinomycetemcomitans* from the subgingival plaque of 35 DS children compared with 35 sex- and age-matched HC. Compared to HC, DS subjects reported a significantly higher prevalence of *A. actinomycetemcomitans* (54% vs. 6%) and worst PI and GI. Moreover, neutrophil chemotaxis in DS was significantly impaired and directly associated with PI and GI, as well as *A. actinomycetemcomitans* levels. Similar findings were confirmed in 2012 by El Housseiny [71] who compared the presence of *A. actinomycetemcomitans* in 60 DS children and 60 normal children, sex- and age-matched. The occurrence of *A. actinomycetemcomitans* was statistically higher in DS children than HC (55% vs. 28%, respectively), without any statistical difference according to age.

In 1999, Agholme et al. [62] reported the results from a longitudinal study on DS, related to the frequency of periodontitis and bacterial prevalences (*A. actinomycetemcomitans*, *Capnocytophaga*, and *P. gingivalis*). From baseline to the endpoint seven years later, the percentage of DS with periodontal pockets significantly increased from 41% to 65%, as well the presence of bone loss, which doubled from 35% to 74%. Conversely, the gingival bleeding prevalence significantly decreased from 67% to 44%. The bacteria occurrences did not significantly differ during the period of the study, and the baselines were 32% (*A. actinomycetemcomitans*), 100% (*Capnocytophaga*), and 3% (*P. gingivalis*). The bacterial presence was not statistically associated with alveolar bone loss, except for Aa, more frequently found in sites with alveolar bone loss.

In 2000, Amano et al. [63] examined the prevalence of periodontal diseases and related periodontopathic bacteria in 60 HC children and 60 DS children, sub-grouped by age, to determine if specific periodontopathogens (*A. actinomycetemcomitans*, *P. gingivalis*, *T. forsythia*, *T. denticola*, *P. intermedia*, *Prevotella nigrescens*, *Capnocytophaga ochracea*, *C. sputigena*, *C. rectus* and *E. corrodens*) are acquired in their childhood. The authors reported signs of gingivitis in 57% of DS and 52% of HC, in both cases increasing with age but without a significant difference, while periodontal pockets were absent in all the 120 subjects studied. The bacteria investigated were always most frequently detected in the DS than in age-matched HC of any age group, except for *P. intermedia*, found slightly higher in HC aged 8–10 years. In detail, in the younger children (2–7 years old), the predominating and significantly higher bacteria found in the DS group than controls were *E. corrodens*, *C. rectus*, *C. sputigea* and *C. ochracea* (present in more than 90% of DS subjects), followed by frequency, by *A. actinomicetemcomitans* (in more than 70% of DS subjects) and *P. nigriscens* (40%). *T. denticola*, *T. forsythia* and *P. gingivalis* were exclusively reported only in DS subjects, with approximative percentages of 42%, 58% and 30%, respectively, increasing with age, while *P. intermedia* detection lacked in both groups at the age of 2–4 years and was reported only in 20% of the DS at the age of 5–7 years. In the age ranges 8–13 years, each bacterium increased its relative prevalence in both groups of children; *T. forsythia* was detectable also in HC in percentages three times lower than in the DS group, while *T. denticola* and *P. gingivalis* were still exclusively reported only in the DS children. Furthermore, *T. forsythia* and *P. gingivalis* were mainly found in subjects with gingivitis, whereas the distribution of *A. actinomycetemcomitans*, *C. ochracea* and *C. sputigena* did not show any relationship to the gingival inflammatory state.

In the same year, Figueiredo et al. [64] investigated the relationship between the gingival and periodontal health status and periodontopathogens (*P. gingivalis*, *T. denticola*, and *T. forsythia*) in 50 subjects with mental disabilities: 25 with DS and 25 with mental retardations (MR) age- and sex-matched. The detection mode was based on the reaction of subgingival plaque to the BANA test card, which is composed of a strip impregnated with benzoyl-DL-arginine Naphthylamide (BANA), and whose color turns blue in the presence of the bacteria investigated, with intensity proportional to their quantity, thus allowing the establishment and quantification of the presence of these bacteria in the subgingival plaque. The considered bacteria have enzyme activity capable to hydrolyze BANA, and the reaction is visually reported on a scale related to the intensity of blue concentration. Results from the BANA-test were then correlated with a series of periodontal parameters. The authors reported no significant differences in the periodontal and gingival health between groups, which were all equally united by plaque accumulation, bleeding on probing (80% of whole subjects) and depth of pockets >3 mm in 25% of DS and 19% of MR subjects. In both groups, a mean of 55% of subjects was positive to the BANA test, which was significantly associated with the pocket depth and bleeding on probing.

In 2000, Hanookai et al. [65] investigated and compared the local distribution of a series of periodontopathogen bacteria and viruses (EBV, HCMV and HSV) in 19 DS subjects, comparing their expression according to the depth of pockets, classified into shallow (1–3 mm deep) and deepest (5–8 mm deep). The 19 DS subjects were randomly recruited blind to periodontal status. The investigated periodontopathogen bacteria were *P. intermedia*, *T. forsythia*, *Capnocytophaga* spp., *P. gingivalis*, *A. actinomycetemcomitans*, *C. rectus*, *Fusobacterium* spp., *Staphylococcus* spp., *Enterobacteriaceae* spp., *Pseudomonadaceaea* spp., and identified with cultural methods as well as for Candida spp., while the viruses researched were EBV, HCMV and HSV. All 19 DS showed periodontitis of various degrees (mild in 16% of DS, moderate and severe in 42%). The periodontopathogen bacteria were significantly detected in higher proportion at the deepest pockets; the significantly higher at those sites were *P. intermedia*, *T. forsythia*, *Capnocytophaga* spp. and *A. actinomycetemcomitans*. A similar condition was also reported for viruses: those mainly detected at the deepest pockets were, in decreasing prevalences, EBV in 32%, HCMV in 26%, HSV in 16% and viral co-infections in 11%. Candida spp. presence was low in all subjects and did not statistically differ according to the depth of the pocket.

In 2001, Amano et al. [66] investigated the differences in the prevalence of a series of periodontopathogen bacteria in the subgingival plaque in a group of 67 DS and one group of 41 age-matched MR, both with poor oral hygiene and plaque index [80] >80%. All subjects reported BOP+ and mean GI >= 1. Gingivitis was found in 42% of DS and in 76% of MR, while periodontitis affected the remaining 58% of DS (36% with shallow depth of pockets and 22% with deepest pockets) and 24% of MR (19.5% with shallow depth of pockets and 4.5% with deepest pockets). The DS group reported a significantly higher prevalence of marginal periodontitis compared with the MR group. Twenty-eight DS reported gingivitis (G-DS) and the other 39, periodontitis (P-DS, 24 with shallow depth of pockets and 14 with deepest pockets). The authors did not report any significant difference between groups in the occurrences of bacterial species, but, when sub-dividing each group according to periodontal status (subjects with gingivitis and those with periodontitis), some significant differences emerged. The relative prevalences of *P. gingivalis*, *T. forsythia* and *P. intermedia* were significantly higher in the P-DS, compared to the G-DS group. In the subjects carrying *P. gingivalis*, the authors also investigated the genotyping of the gene fimA, a gene encoding fimbrillin (fimA), the structural subunit protein of fimbriae of *P. gingivalis*, involved in the bacterial–host interaction and considered a key factor for *P. gingivalis* periodontopathogenicity; the variant fimA type II has been associated with severe periodontitis [85]. The G-DS group reported the predominant presence of fimA type I (72%), followed by type II (14%) and mixed type I and II in another 14% of subjects. The P-DS group reported a significant prevalence of fimA type II (46%), followed by multiple fimA types variously expressed whose type II predominated (in 20% of coexistence cases). The fimA type I was the most prevalent gene type also in G-MR, followed by type III, while type II confirmed its higher prevalence in the MR-periodontitis group. In both groups, the occurrence of type II fimA genotype was significantly higher in subjects with periodontitis, both DS and MR.

The same research group, one year later in 2002 [69] focused further work on the fimA gene variants in disabled subjects with periodontitis and identified a novel gene variant called fimA type Ib, which was reported strongly expressed in *P. gingivalis* from both DS and MR with periodontitis (P-DS and P-MR, respectively), and correlated with the progression of the disease. The researchers considered *P. gingivalis* prevalence and typing for the gene fimA in 380 healthy subjects without periodontitis (HC), 192 healthy subjects with periodontitis (P-HC), 44 with DS and 39 with MR. In HC, *P. gingivalis* was detected in 36% of the 380 subjects. Most of them (72.5%) had a single fimA genotype: 50% fimA type I, 12% type V, 3.6% type II, 2.9% type Ib, 2.2% type III, 0.7% type IV. The remaining subjects reported more than one type of fimA. Compared to the 192 HC with periodontitis, fimA type I and V were significantly more prevalent in healthy subjects, while type II, Ib and IV were more prevalent in the periodontitis group. In the disabled population, *P. gingivalis* was found in 66% of DS and in 71% of MR, with a significant occurrence of fimA type Ib (17%) and type II (47%) in P-DS, while in G-DS (DS subjects with gingivitis), fimA type I was statistically predominant (71%), followed by types Ib and II (14%). In the 71% of MR positive to *P. gingivalis*, the fimA types that occurred were type II (47%) and type Ib (17%) in P-MR (mentally retarded persons with periodontitis) and type I (33%) and III (19%) in G-MR (mentally retarded persons with gingivitis). FimA type Ib was associated with periodontitis with statistical relevance only in the MR group, and not in the DS subjects.

A third paper investigated the fimA gene typing in DS with and without periodontitis. The work was a cross-sectional study performed on 75 DS by Martinez-Martinez et al. in 2013 [73]. Of the 75 DS, 45 with periodontitis (P-DS) and 30 without periodontitis (H-DS) were considered to characterize four periodontal bacteria in both groups (*P. gingivalis*, *T. denticola*, *T. forsythia* and *A. actinomycetemcomitans*), as well as the fimA genotypes expressed in *P. gingivalis* infections. The total amount of subjects positive to the presence of the three red-complex bacteria was statistically higher in the P-DS than in H-DS (47% vs. 17%), while, conversely, H-DS reported more frequently lack of all of them (20%) or *T. forsythia* alone (20%). *T. forsythia* was the most frequently reported species (95.5% in P-DS and 63.3% in H-DS), followed by *T. denticola* (88.8 and 50%) and *P. gingivalis* (53.3 and 25%, respectively). With regards to the fimA genotypes from *P. gingivalis* in P-DS, the type I was the most frequently reported (in 75% cases), followed, by frequency by type II (54%), type IV (17%), type Ib (12.5%) and type III (8.3%). Otherwise, in H-DS, the most frequently reported fimA was type II (75%), followed by types I and IV (both 37.5%), and type Ib (25%); no fimA type III and V were reported in these subjects. Some samples, mainly from P-DS, were positive for multiple fimA alleles indicating that various genotypes of *P. gingivalis* were present. Furthermore, loop-mediated amplification method (LAMP) was used in case of positivity to *A. actinomycetemcomitans* to recognize its variant JP2, strongly associated with severe aggressive periodontitis due to its proven potentiated leukotoxin activity [86].

In 2001, Reuland-Bosma et al. [67] contrasted the previous evidence by reporting no significant differences in clinical gravity of periodontitis and in periodontopathogen prevalence in the subgingival plaque of 17 DS compared with 17 age-matched controls, with the exception of *P. gingivalis*, which was significantly higher in the DS group. The mean number of tooth loss and the pocket depth in DS subject were statistically higher than in HC, while, within the DS subjects, no statistical differences were reported between DS at low risk and high risk of periodontitis (risk was established according to the number of teeth lost for periodontitis, at the cut off of six teeth), although *P. gingivalis* was more prevalent in the “low risk” group and *F. nucleatum* in the “high risk” one.

In 2001, Sakellari et al. [68] reported the result from a longitudinal study on five DS with periodontitis to evaluate the microbiota composition of the supra- and subgingival plaque monitored at the baseline and during the treatments over a period of 5 years. Although the periodontal indices improved at the endpoint, plaque levels dropped from 100% of the sites at the baselines, to remained still stably high, at 60%, at the endpoint. All periodontopathogen bacteria investigated were detected at all time points, both supragingivally and subgingivally with different associations. In detail, at the baseline, *P. gingivalis* was reported in some cases only subgingivally, contrary to *C. sputigena e S. oralis*, present only supragingivally. After 1 month from treatment, the percentage of bacterial detection significantly decreased for *C. rectus*, *E. corrodens*, *Actynomices naeslundii* and *Peptostreptococcus micros* supragingivally, and subgingivally the same species were detected, plus *P. gingivalis*, *T. forsythia*, *F. nucleatum* and *S. oralis*. Three months after scaling and root planing, the percentages of detection were reduced further. *C. rectus*, *F. nucleatum*, *E. corrodens*, *P. micros*, *V. parvula* and *A. naeslundii* were reduced significantly both supragingivally and subgingivally (*p* = 0.05). A significant reduction was noticed for *P. gingivalis* and *C. sputigena* supragingivally, while *A. actinomycetemcomitans*, *T. forsythia*, *P. intermedia* and *P. nigrescens* significantly decreased subgingivally. Six months after treatment, the percentages of detection increased for most species. Significant increases were particularly noticed for *F. nucleatum*, *P. micros* and *V. parvula* both supragingivally and subgingivally, *T. forsythia* and *C. sputigena* only supragingivally, and *E. corrodens* and *Streptococcus sanguis* only subgingivally.

Four years later, the same research group [70] reported the results from a cross-sectional case-control study on 70 DS, 121 systemically healthy subjects (HC) and 76 individuals with cerebral palsy (CP) to correlate the periodontal conditions and the subgingival microbiota among the groups. Subjects were sub-classified, according to age, into children, adolescents and young adults, and any clinical and microbiological differences between them were investigated. Clinical indices of PDs and treatment needs were statistically significantly higher among DS at any age, compared with the other two groups. In detail, DS reported significantly higher percentages of deep pockets and higher loss of attachment, mainly in adolescents and young adults, when compared with age-matched HC and CP. With regards to subgingival periodontopathogen bacteria, in DS compared with HC and CP, *T. forsythia* and *A. naeslundi* were the species significantly more prevalent at any age; *P. gingivalis*, *A. actinomycetemcomitans*, *C. rectus*, *P. intermedia*, and *C. sputigena* were more prevalent in adolescents and young adults; *E. corrodens*, *P. nigrescens* and *P. micros* were peculiarly significative higher in DS young adults.

In 2012, Khocht et al. [72] compared the subgingival microbiota in a series of 44 DS, 83 HC and 66 MR by checkerboard DNA–DNA hybridization. Per each group, the authors distinguished subjects with and without periodontitis. The percentages of subjects with periodontitis within each group did not differ statistically, and were 72% for the DS group, 71% for the MR group and 78% for the HC. Furthermore, the P-DS group showed statistically worst clinical periodontal parameters such as higher loss of attachment and number of pockets deeper than 5 mm, compared with MR, and higher number of missing teeth, gingival index and plaque index scores than HC and MR. Among the 40 periodontopathogen species investigated, some of the yellow complex (*Selenomonas noxia*, *Propionibacterium acnes*, *Streptococcus gordonii*, *Streptococcus mitis* and *Streptococcus oralis*) were significantly higher in DS than in HC and MR; *Streptococcus constellatus* (orange complex) was higher in DS than HC; *Treponema socranskii* was significantly higher in DS than in MR and, particularly in the P-DS group. Furthermore, in P-DS, higher levels of *S. constellatus*, *F. nucleatum ssp.nucleatum*, *P. nigrescens* (orange complex) and *S. noxia* showed significant positive correlations with attachment loss, while *A. naeslundii* and *Actinomyces odontolyticus* were negatively associated with this periodontal parameter.

In 2014, Ahmed et al. [74] from India compared and quantified the presence of two periodontal pathogens—*A. actinomycetemcomitans* and *P. gingivalis* in P-DS, G-DS, P-HC, and G-HC. The results reported statistically significant higher levels of both bacteria in DS compared with HC, both in the case of gingivitis and periodontitis. *P. gingivalis* was particularly and significantly higher in G-DS than in G-HC, while *A. actinomycetemcomitans* significantly higher in P-DS than P-HC. Similarly, four later, Ahmed et al. [78] reported significantly higher levels of *T. forsythia* and *T. denticola* in 20 P-DS compared with 20 P-HC and 20 HC without periodontitis.

In 2015, Mehr et al. [75] reported the prevalence of the parasite *Trichomonas tenax* in the oral cavity of 52 DS children with PDs and further 52 sex- and age-matched HC, without PDs. The researchers found a statistically higher occurrence of the infection of *T. tenax* in 27% of DS subjects compared with 9.6% of HC, and a significantly higher gingival index in DS than HC, while the plaque indices did not differ significantly.

In the same year, Tanaka et al. [76] studied the clinical effects and microbiological changes in a series of 35 subjects affected by chronic periodontitis: the case group consisted of 23 DS subjects, the control group by 12 healthy subjects. At the baseline, the P-DS group reported significantly higher levels of plaque than the P-HC group (84% vs. 48%, respectively) and gingival bleeding (44% in P-DS vs. 26% in P-HC). At the end of periodontal mechanic treatment, both groups improved in the clinical parameters of diseased sites but revealed no statistical differences between groups. Otherwise, the microbial composition was reported significantly different. Before treatment, at diseased sites, the levels of *P. gingivalis* and *T. forsythia* were similar in both groups, while *T. denticola* levels were found significantly higher in P-DS; at the healthy sites, *P.gingivalis* was significantly higher in P-DS subjects. Forty-five days after the completion of mechanical treatment (consisting of oral hygiene instruction, scaling, and root planing under local anesthesia), the levels of the bacteria investigated decreased in both groups, although in the P-DS they were still significantly higher than in P-HC, revealing that the non-surgical periodontal therapy did not significantly reduce the bacterial counts of the three investigated micro-organisms in the diseased sites of the P-DS group, contrary to the P-HC.

In 2016, Faria-Carrida et al. [77] compared periodontal status, types and quantity of a series of eight periodontopathogen bacteria (*C. rectus; P. gingivalis; T. denticola; F.nucleatum; P. intermedia; P. nigrescens; A. actinomycetemcomitans; T. forsythia*) in 30 DS children and 30 HC, by FISH (fluorescence in situ hybridization). All eight species were reported in both groups, with a mean of 80% in the HC and with percentages close to 100% in the DS group. The species significantly higher in the DS group than in the HC group were *C. rectus*, *P. gingivalis*, *T. denticola*, *F. nucleatum*, *P. intermedia* and *P. nigrescens.* According to age, DS in the range of 3–7 years reported significant higher bacterial density for *F.nucleatum*, *P.intermedia*, and *P.nigrescens* (orange complex), while, in the age range 8–12, only *C.rectus* density was found significantly higher in the DS group. Gingivitis was significantly more frequent in the DS group (37% vs. 13% of HC subjects), while the PI score did not significantly differ.

**Figure 3:**
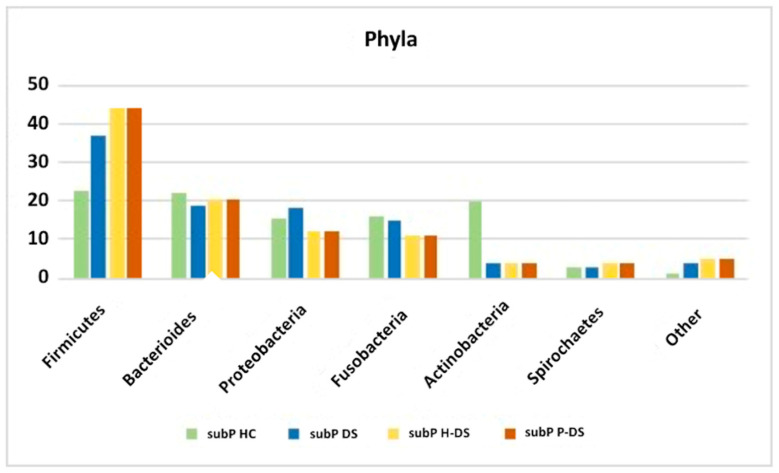
The most represented phyla, as reported form Segata et al. [48] for subgingival plaque of HC (subP HC), and from Novoa et al. [52], for subgingival plaque of DS, irrespective of periodontal status (subP DS), and in those with (subP P-DS) and without periodontitis (subP H-DS).

In 2020, Novoa et al. [52] performed a study with NSG (next-generation sequencing) for the total bacterial genomic DNA identification in the subgingival plaque from 25 P-DS, compared with 25 H-DS. The authors showed shown significant differences between the oral microbiome of the two groups at various taxonomical levels. In detail, the operational taxonomic units (OTUs) of bacteria residing in the oral cavity were classified into 13 phyla, of whom the most represented, in the total 50 DS subjects and according to periodontal status, have been reported in Figure 3, which compare the percentages with those found by Segata et al. [48] in the pioneering work on normal oral microbiota composition in healthy subjects.

Concerning phyla reported from Novoa et al., significant differences between P-DS and H-DS were found in the relative percentages of *Proteobacteria* (higher in H-DS than P-DS), and *Synergistetes* and *Chloroflexi* (higher in P-DS than in H-DS). Significant differences were remarkably strong also at genus and species levels. At genus level, while *Streptococcus*, *Fusobacterium*, and *Neisseria* were the genera most represented in both groups, *Pseudomonas* and *Veillonella* were more represented in the H-DS group than in the P-DS group, and, conversely, *Porphyromonas* and *Treponema* (4.10%) prevailed in the P-DS group. Among the 21 genera significantly differing between groups, *Tannerella* was significantly more abundant in the P-DS, and *Pseudomonas* in the H-DS. At species levels, among the 403 species identified, Novoa et al. reported peculiar and significantly different relative abundances of 39 species between groups. While *Streptococcus* spp. and *Fusobacterium* spp. were the most abundant species in both groups, other species statistically differed. In detail, *Pseudomonas* spp., *Granulicatella* spp., *Veillonella* spp. and *Gemella* spp. were the most abundant in the H-DS group, and *Porphyromonas* spp., *Treponema* spp., *Aggregatibacter* spp. and *Tannerella* spp. were the most abundant in P-DS. The P-DS group also harbored higher levels of newly described putative pathogens, such as *Fretibacterium*, *Peptostreptococcus*, *Desulfobulbus* and *Filifactor*.

One year later, the same authors reported further findings in another study on subgingival microbiota of DS, by approaching its identification via cultural methods and q-PCR (quantitative polymerase chain reaction) [15]. 164 DS were stratified into 62 H-DS, 34 G-DS and 28 P-DS. P-DS were significantly older than those of the other two groups. By q-PCR, the frequency of detection and counts of *A. actinomycetemcomitans* were low in all groups, although, together with *P. gingivalis*, its frequencies and counts increased—not significantly—in P-DS. *T. forsythia* was the most prevalent species in all groups (67.9% in P-DS, 47.0% in G-DS and 48.8% in H-DS), with higher significant percentages in P-DS when compared with G-DS and H-DS. Significantly higher counts of total anaerobic bacteria were reported in P-DS, when compared with H-DS. *P. gingivalis*, *P. intermedia*, *T. forsythia* and *E. corrodens* progressively increased in frequency of detection, counts and proportions, parallel with the worsening of the periodontal status. Conversely, *P. micra* and *A. odontolyticus* were found higher in H-DS, while *C. rectus* and *Capnocytophaga* spp. presented the highest counts, proportions and frequencies in G-DS.

The last and more recent work considered in the present systematic review is the one from Willis et al. [51]. The authors focused on the oral microbiome and—for the first time in the literature—also on the mycobiome characterization in 27 DS compared with their relatives, HC. The authors adopted several methods to gain the more precise results in microbial finding. NGS and PCR were used for discovering taxonomy assignment of bacteria from their 16S RNA peculiar sequences according to the library from the Human Microbiome Project [87,88]. The results proved that, compared to their healthy relatives, DS showed higher abundance of bacteria from the genera *Kingella*, *Gemella*, *Cardiobacterium*, *Staphylococcus*, *Rothia* and *Actinobacillus*, and lower abundance of *Alloprevotella*, *Atopobium* and *Candidatus Saccharimonas genera.* Fungal identification was performed by culture-based methods coupled with proteomics. On this regard, in DS, a more prevalent proportion of fungi than in relative controls was reported (54% vs. 26%), with a higher and significant prevalence of opportunistic pathogens as *C. parapsilosis* (in 15.4 % of DS vs. 0.60 % of HC) and *C. dublinienis* (15.4% vs. 1.51%), while *C. albicans* prevalence was 30.8% in DS and 19% in HC.

## 4. Discussion and Conclusions

The present systematic review aimed to report and describe the pieces of evidence in the scientific literature about the possible peculiarities of the oral microbiota of subjects affected by Down syndrome, with and without periodontal diseases, and compare the data with healthy subjects not affected by DS. For this purpose, a series of 26 heterogeneous works were selected, published from 1968 to 2021.

The choice to perform the search without year limitations in the last decades was for “historical purposes”: thanks to this criterium, it has been possible to follow the storyline of the discoveries focusing on periodontal diseases in DS, and to understand how, from a culture-based to a culture-independent approach, substantial signs of progress of knowledge about, not only the species but also their variants, can contribute to better understand the peculiarities of the DS microbiome.

Most of the works compared DS with and without PDs, or DS with HC, or with subjects affected by other mental disabilities, to measure the weight of the motor and cognitive disabilities in the early and aggressive onset of PDs in DS and to elicit any possible differences in the onset and features of PDs and in microbial composition in DS compared with MR and CP. In detail, Sakellari et al. in 2005 [70] demonstrated that DS have significant worst clinical indices of periodontal diseases than other mentally retarded subjects, as those with cerebral palsy, as well the microbiota composition in term of periodontopathogen bacteria, being *T. forsythia* and *A. naeslundi* significantly more prevalent at any age; *P. gingivalis*, *A. actinomycetemcomitans*, *C. rectus*, *P. intermedia*, and *C. sputigena* more prevalent in adolescents and young adults; *E. corrodens*, *P. nigrescens* and *P. micros* peculiarly significative higher in DS young adults. Similar statistically significative differences were reported by Khocht et al. [72] comparing DS subjects and non-DS with mental retardation, both affected by periodontitis: also, in this case, DS reported worst periodontal indices and a significant increase in periodontopathogen bacteria of the orange and yellow complexes higher in DS than in MR. Therefore, it is reasonable to hypothesize that poor motor skills are not the only cause responsible for the higher prevalence of periodontitis in Down syndrome than in healthy subjects, and more severe and precocious forms in Down syndrome than in those with other non-DS-mentally retarded.

Furthermore, Tanaka et al. [76] reported that the non-surgical periodontal therapy of periodontitis failed to significantly reduce the periodontopathogen bacterial counts in the diseased sites of DS subjects, contrary to the healthy controls.

Fil rouge of most of the works was the age of subjects enrolled. Most part of the studies focused mainly on DS children, being directly involved in gingivitis, and on young adults, being the periodontitis in DS associated with early-onset than in healthy, euploid subjects.

From the qualitative analysis, despite the high and detailed number of researches focusing on bacterial microbiota in DS, only two works focused on fungal and viral compositions. Willis et al. [51] reported the identification of a specific mycobiome in DS subjects compared with non-DS persons, and Hanookai et al. [65] investigated the presence of a series of viruses and *C. albicans* in the deepest pockets of DS subjects with periodontitis. Apart from these two works, a sad lack of extended studies on fungal, viral and parasitic occurrences in the oral microbiome of DS have emerged. This datum should orient research to investigate the occurrence of not only other microbial species, but also viruses, fungi and protozoan.

With stronger data, personalized medicine could be designed to pay more attention to particular and critical conditions as those suffered by the DS subjects.

## Figures and Tables

**Table 1:** The periodontopathogen potential of each bacterial complex of the subgingival plaque, according to Socransky et al., 1998 [45].

**Green complex** *Eikenella corrodens* *Capnocytophaga* spp. (*C. gingivalis*, *C. ochracea*, *C. sputigena*) *Actinomyces actinomycetemcomitans serotype a*	- The “early colonizers” - Moderately periodontopathogen - Adhere with their fimbriae to the pellicle and put the bases for periodontopathogens colonization)
**Yellow complex (orange-associated)** *Streptococcus* spp. (*S.mitis*, *S.sanguis*, *S.oralis*, *S.intermedius*, *S.gordonii*)	
**Orange complex** *Eubacterium nodatum* *Peptostreptococcus micros* *Prevotella intermedia* *Prevotella nigrescens* *Fusobacterium nucleatum* spp (*Fusobacterium nucleatum ss vincentii*, *Fusobacterium nucleatum ss polymorphus*) *Campylobacter* spp. (*C. rectus C. showae*, *C. gracilis*) *Streptococcus constellatus*	- The “bridge species”; are the link between the green and red complexes - Produce toxins and enzymes responsible for progressive attachment loss and increase in the pocket depth - Create a hospitable environment in the gingival sulcus/pocket for the living condition and colonization of the red complex bacteria
**Red complex** Porphyromonas gingivalis Tannerella forsithya Treponema denticola	- The most periodontopathogen species; are significantly involved in the destruction of the periodontium - Gram negative, Anaerobic - Penetrate and aggressively destruct tissues the soft tissue and bone through the production of potent virulence factors.
**Purple complex** *Veillonella parvula* *Actinomyces odontolyiticus*	Not associated with other complexes
**Aa-complex** *Actinomyces Actinomicetemcomitans serotype b* *Actinomyces naeslundii* *Selenomonas noxia*	Do not cluster with other species

**Table 2:** The MeSHs terms used in the search and their combinations used by Boolean connectors.

Search Topic		Search Items (May 2020)
“Down syndrome” OR “21 trisomy” OR “trisomy 21”	AND	“oral microbiome”
		“oral microbiota”
		biofilm
		“periodontal diseases”
		gingivitis
		periodontitis
		gingival
		gingiva
		gums
		periodontium
		periodontal
		“periodontal dysbiosis”
		“oral dysbiosis”
		“gingival dysbiosis”

**Table 3:** Main characteristics of the studies included.

	First Author, (Year), Country	Type of Study	Aim of the Study	n. and Type of Subjects Enrolled #	Mean Age (Range) Yr	Sex F:M (%)	Periodontal Status	Periodontal Parameters *	Sample Origin	Methods of Microbial Identification &	Micro-organisms Investigated $	Report on Association Microbiota/ Periodontal Status	Statistical Analysis
1	Meskin et al. [56].(1968) USA.	Case-control	Prevalence of B.m. and PDs in DS, CP and HC children	41 P-DS	(5–12)	n.r.	Gingivitis+ Alveolar bone loss	n.r.	Gingival plaque	Culture	B.m.	Yes	Yes
				19 G-CP	(5–8)		Gingivitis						
				20 HC	(5–12)		Healthy						
2	Barr-Agholme et al. [57] (1992) Sweden	Case-control	Levels of A.a., Capnocytophaga and P.g. in DS and HC	37 DS	16.3 ± 3.4 (9–21)	17:20 (46:54)	Blind	GBI; calculus; DoP; BL	Subgingival plaque	Culture	A.a., Capnocytophaga and P.g	Yes	Yes
				37 HC									
3	Santos et al. [58] (1996) U.S.A.	Case-control	Levels of circulating antibodies to A.a and association with PDs in DS and HC	5 G-DS	24.0 ± 3.16	n.r.	Gingivitis	DoP	Serum	ELISA #	A.a antibodies	Yes	Yes
				11 P-DS	21.9 ± 7.8		Periodontitis						
				10 HC	21.4 ± 4.7		Healthy						
4	Morinushi et al. [59] (1997) Japan	Case-control	Levels of serum antibodies to periodontopathogens and association with gingivitis in DS	75 DS	(2–18)	28:47 (37:63)	Blind	GBI; OHI	Serum	ELISA #	P.g., P.i., T.d., F.n., A.a. Mi, Sel.s.	Yes	Yes
				300 HC		123:17 (41:59)							
5	Cichon et al. [60] (1998) Germany	Case-control	Periodontal and microbiological status in DS and HC	10 DS	26.3 (20–31)	4:6 (40:60)	Blind	PI; GI; DoP; CAL	Subgingival plaque	DNA probes	A.a., P.g., P.i., T.d., F.n., E.c., T.f., C.r.	Yes	Yes
				11 CP	36 (23–53)	4:7 (36:67)							
6	Sreedevi et al. [61] (1998) India	Case-control	Relationship between the gingival and periodontal health status and Aa in DS and HC	35 DS	(6–14)	16:19 (46:54)	Blind	PI; GI	Subgingival plaque	Culture	A.a.	Yes	Yes
				35 HC	Age-matched	Sex-matched							
7	Agholme et al. [62] (1999) Sweden	Longitudinal cohort	Development of PD during 7 years in DS	37 DS	16.6 ± 3.6 (baseline) 23.5 ± 3.4 (endpoint)	16:18 (43:57)	Blind	GBI; DoP	Subgingival plaque	Culture	A.a., Capnocytophaga, P.g.	Yes	Yes
8	Amano et al. [63] (2000) Japan	Case-control	Prevalence of periodontopathogens in DS children	60 DS	(2–13)	26:34 (43:57)	Blind	BOP; DoP; GI	Subgingival plaque	PCR	A.a., P.g., T.f., T.d., P.i., P.n., C.o., C.s., C.r., E.c.	Yes	Yes
				60 HC	(2–13)	28:32 (47:53)							
9	Figueiredo et al. [64] (2000) Brazil	Case-control	Relationship between the gingival and periodontal health status and periodontopathogens in DS and MR	25 DS	23.3 ± 7.1 (12–33)	10:15 (40:60)	Blind	BOP; PBS; DoP; PI	Subgingival plaque	BANA test card	P.g., T.d., T.f.	Yes	Yes
				25 MR	20.9 ± 7.3 (15–39)	11:14 (44:56)							
10	Hanookai et al. [65] (2000) USA.	Cohort	Occurrence of herpesviruses and periodontopathic bacteria in H-DS and P-DS	19 DS	23.7 ± 7.5 (17–37)		Blind	PI; GI	Subgingival plaque	PCR (viruses), culture (bacteria)	EBV, HCMV, HSV, P.i., T.f., Capnocytophaga spp., P.g. A.a., C.r., Fusobacterium spp.; Staphylococcus spp.; Enterobacteriaceae spp.; Pseudomonadaceaea spp. and Candida Spp.	Yes	Yes
11	Amano et al. [66] (2001) Japan	Case-control	Bacterial prevalence in subgingival plaque samples from DS and MR	67 DS	25.40 ± 4.88 (20–35)	32:35 (48:52)	Subjects with poor oral hygiene (PI > 80%)	PI; DoP; BOP; GI	Subgingival plaque	PCR	A.a., P.g., T.f., T.d., P.i. P.n., C.o., C.s., C.r., E.c.; fimA genotypes identification in P.g.-positive samples.	Yes	Yes
				41 MR	27.00 ± 5.05 (20–35)	19:22 (46:54)							
12	Reuland-Bosma et al. [67] (2001) Netherlands	Case-control	Identification of the subgingival microflora in DS and MR	17 DS	(31.5–44.5)	n.r.	DS with low and high risk periodontits	PI; BOP; DoP	Subgingival plaque	Culture	A.a., P.g., P.i., T.f., P.m., F.n. C.r.	Yes	Yes
				17 MR	(32.0–47.5)	n.r.	Blind						
13	Sakellari et al. [68] (2001) Greece	Longitudinal cohort	Supragingival and subgingival microbiological status according to periodontal treatment	5 P-DS	(26–37)	2:3 (40:60)	Periodontitis	DoP; BOP; PI	Supra- and subgingival plaque	DNA probes	A.a., P.g., P.i.,P.n., T.f., F.n., V.p., C.r., P.m., E.k., C.s., S.s., S.o., An	Yes	Yes
14	Nakagawa et al. [69] (2002) Japan	Case-control	P.g. fimA typization according to systemic and periodontal conditions in DS. MD, and HC	380 HC	(30–70)	217:163 (57:43)	Healthy	n.r.	Supra- and subgingival plaque	PCR	fimA type Ib	Yes	Yes
				192 P-HC	(30–70)	89:103 (46:54)	Periodontitis						
				44 DS	25.4 ± 4.88 (20–35)	21:23 (48:52)	Blind						
				39 MR	27.0 ± 5.05 (20–35)	15:14 (39:61)	Blind						
15	Sakellari et al. [70] (2005) Greece	Case-control	Periodontal conditions and subgingival microflora in DS, HC and CP	70 DS	8–28	n.r.	Blind	PI; DoP; BOP; GI	Subgingival plaque	DNA probes	P.g., A.a., T.f., P.i., P.n., F.n., C.r. V.p. P.m., E.c., C.s., S.s., S.o., A.n.	Yes	Yes
				121 HC									
				76 CP									
16	El Housseiny [71] (2012) Saudi Arabia	Case-control	A.a. in DS and HC children	60 DS	10.20 ± 3.06 (5–14)	(52:48)	Blind	Not considered	Subgingival plaque	Culture	A.a.	No	Yes
				60 HC	9.39 ± 2.89 (5–14)	(44:56)							
17	Khocht et al. [72] (2012) USA.	Case-control	Subgingival microbiota of DS, MR and HC	12 H-DS 32 P-DS	35.81 ± 1.83 (18–56)	(52:48)	30% healthy, 30% with periodontitis per each group	GI; PI; DoP	Subgingival plaque	DNA probes	40 bacterial species	Yes	Yes
				19 H-MR 47 P-MR	46.15 ± 1.40 (22–84)	(42: 58)							
				17 HC 66 P-HC	40.93 ± 1.33 (18–73)	(55:45)							
18	Martinez-Martinez et al. [73] (2013) Mexico	Case-control	Characterization of the main periodontal bacterial species in H-DS and P-DS	45 P-DS	24.7 ± 7.7	15:30 (33:67)	Periodontitis	DoP; CAL	Subgingival plaque	PCR, LAMP	P.g. (and its fimA genotypes); A.a. (and its variant JP2); T.d., T.f.	Yes	Yes
				30 H-DS	21 ± 4.3 years	13:17 (43:57)	Healthy						
19	Ahmed et al. [74] (2014) India	Case-control	Levels of A.a. and P.g. in G-DS, P-DS, G-HC, P-HC	18 G-DS	18.4 ± 2.2	14:15 (48:52)	Gingivitis	BOP; DoP; CAL; GI	Subgingival plaque	RT-PCR	A.a., P.g.	Yes	Yes
				11 P-DS			Periodontitis						
				15 G-HC	21.3 ± 3.2	12:18 (40:60)	Gingivitis						
				15 P-HC			Periodontitis						
20	Mehr et al. [75] (2015) Iran	Case-control	Prevalence of T.t. in oral cavity of P-DS and HC	52 P-DS	(5–12)	24:28	PDs	PI; GI (L&S)	Subgingival plaque	PCR	Trichomonas tenax (parasite)	Yes	Yes
				52 HC	(5–12)	31:21	Healthy						
21	Tanaka et al. [76] (2015) Brazil	Case-control	Clinical and microbiological parameters according to treatment in P-DS and P-HC	23 P-DS	31.91 ± 5.85	8:15 (35:65)	Periodontitis	DoP; CAL; BOP	Subgingival plaque	qPCR	P.g., T.d., T.f.	Yes	Yes
				12 P-HC	41.25 ± 6.17	5:7 (42:58)	Periodontitis						
22	Faria Carrada et al. [77] (2016) Brazil	Case-control	Sssessment of salivary periodontopathic bacteria in DS and HC	30 DS	6.37 ± 2.50 (3–12)	13:17 (43:57)	Blind	GBI; PI	Unstimulated saliva	FISH	C.r., P.g., T.d., F.n., P.i., P.n., A.a, T.f.	No	Yes
				30 HC	7.53 ± 2.15 (4–12)	16:14 (56:44)							
23	Ahmed et al. [78] (2018) India	Case-control	Levels of T.d. and T.f in P-DS, HC, and P-HC	20 P-DS	n.r.	n.r.	Periodontitis	OHI; PI	Subgingival plaque	PCR	T.f., T.d.	Yes	Yes
				20 P-HC			Periodontitis						
				20 HC			Healthy						
24	Novoa et al. [52] (2020) Spain	Case-control	Subgingival microbiome in DS	25 P-DS	28.6 ± 6.61	10:15 (40:60)	Periodontitis	PI; DoP; BOP; CAL	Subgingival plaque	NGS	TOTAL GENOMIC DNA	Yes	Yes
				25 H-DS	25.1 ± 6.18	11:14 (44:56)	Healthy						
25	Cuenca et al. [15] (2021) Spain	Case-control	Subgingival microbiome in DS	62 H-DS	21.4 ± 7.5 (6–40)	27:35 (44:56)	Healthy	PI; DoP; BOP; CAL	Subgingival plaque	Culture, qPCR	PCR: A.a., P.g., T.f. culture: A. a., Capnocytophaga spp., C.r. E.c., F.n., P.g., P.i., P.micra T.f., A.o.	Yes	Yes
				34 G-DS	22.6 ± 6.0 (11–34)	18:15 (54:56)	Gingivitis						
				28 P-DS	27.9 ± 7.2 (10–42)	10:18 (36:64)	Periodontitis						
26	Willis et al. [51] (2020) Spain	Case-control	Microbiome and mycobiome in DS and HC	27 DS	(7–33)	n.r.	Blind	Not considered	Saliva from mouthwash	NGS, PCR, culture and proteomics	NGS, PCR: bacteria. culture and proteomic: Candida spp.; Molds; bacteria	No	Yes
				20 HC	(7–77)	n.r.							

Legend: **# Subjects enrolled and periodontal status: MR,** subjects with mental retards; **DS,** subjects with Down syndrome; **H-DS,** periodontally healthy subjects with Down syndrome; **P-DS,** Down syndrome subjects with periodontitis; **G-DS,** Down syndrome subjects with gingivitis; **HC,** healthy controls, without DS; **P-HC,** healthy controls with periodontitis; **G-HC,** healthy controls with gingivitis; **MR,** subjects with mental retardation; **CP,** subjects with cerebral palsy, without DS; **G-CP,** subjects with cerebral palsy and gingivitis; **PDs,** periodontal diseases. * Periodontal parameters: PDs, periodontal diseases not otherwise specified; **BL,** alveolar bone loss; **BOP,** bleeding on probing; **CAL,** clinical attachment level; **DoP,** depth on probing; **GBI,** gingival bleeding index; **GI,** gingival index; **OHI,** oral hygiene index (plaque score); **PBS,** papillary bleeding score; **PI,** plaque index. **& Methods of microbial identification: ELISA,** enzyme-linked immunosorbent assay; **FISH,** fluorescence in situ hybridization; **LAMP,** loop-mediated isothermal amplification; **PCR,** polymerase chain reaction. **$ Micro-organisms investigated:** **A.a,** *Actinobacillus actinomycetemcomitans*; **A.n.,** *Actinomyces naeslundii*; **A.o.,** *Actinomyces odontolyticus*; **B.m.,** *Bacteroides melaninogenicus*; **C.o.,** *Capnocytophaga ochracea*; **C.r.,** *Campylobacter rectus*; **C.s.,** *Capnocytophaga sputigena*; **EBV,** Epstein Barr Virus; **E.c.,** *Eikenella corrodens*; **F.n.** *Fusobacterium nucleatum*; **HCMV,** *Human cytomegalovirus*; **HSV,** Herpes simplex virus; **Mi,** *Streptococcus mitis*; **P.g.,** *Porphyromonas gingivalis*; **P.i.,** *Prevotella intermedia*; **P.micra,** *Parvimonas micra*; **P.m.,** *Peptostreptococcus micros*; **P.n.,** *Prevotella nigriscens*; **Sel.s.** *Selenomonas sputigena*; **S.o,** *Streptococcus oralis*; **S.s.** *Streptococcus sanguis*; **T.d.,** *Treponema denticola*; **T.f.** *Tannerella forsythia* (formerly *Bacteroides forsythus*); **T.t.,** *Trichomonas tenax*; **V.p.,** *Veillonella parvula*.

**Table 4:** Risk of bias assessment for each study.

First Author (Year)	Bias Due to Confounding	Bias in Participant Selection	Bias in Classification of Interventions	Bias Due to Departure from Intended Intervention	Bias Due to Missing Data	Bias in Measurement of Outcomes	Bias in Selection of the Reported Result	Overall Bias
Meskin et al. [56] (1968)	UR	MR	MR	MR	LR	LR	LR	MR
Barr-Agholme et al. [57] (1992)	UR	SR	SR	MR	LR	LR	MR	SR
Santos et al. [58] (1996)	MR	MR	LR	MR	MR	LR	MR	MR
Morinushi et al. [59] (1997)	UR	MR	MR	MR	LR	LR	LR	MR
Cichon et al. [60] (1998)	UR	MR	MR	LR	MR	LR	MR	MR
Sreedevi et al. [61](1998)	MR	MR	LR	MR	MR	LR	MR	MR
Agholme et al. [62] (1999)	UR	MR	MR	UR	MR	MR	MR	MR
Amano et al. [63] (2000)	UR	MR	LR	LR	LR	LR	LR	MR
Figueiredo et al. [64] (2000)	UR	MR	MR	MR	MR	LR	MR	MR
Hanookai et al. [65] (2000)	UR	LR	MR	LR	LR	LR	LR	LR
Amano et al. [66] (2001)	UR	MR	MR	MR	MR	MR	LR	MR
Reuland-Bosma et al. [67] (2001)	MR	MR	MR	MR	MR	LR	LR	MR
Sakellari et al. [68] (2001)	UR	MR	MR	MR	LR	LR	LR	MR
Nakagawa et al. [69] (2002)	SR	MR	SR	MR	MR	MR	LR	MR
Sakellari et al. [70] (2005)	UR	LR	MR	MR	MR	LR	LR	MR
El Housseiny [71] (2012)	UR	LR	MR	MR	MR	MR	MR	MR
Khocht et al. [72] (2012)	UR	SR	MR	MR	MR	MR	LR	MR
Martinez-Martinez et al. [73] (2013)	MR	SR	MR	MR	MR	SR	MR	SR
Ahmed et al. [74] (2014)	UR	MR	MR	MR	MR	MR	LR	MR
Mehr et al. [75] (2015)	UR	SR	MR	MR	MR	MR	LR	MR
Tanaka et al. [76] (2015)	UR	LR	MR	MR	MR	MR	LR	MR
Faria Carrada et al. [77] (2016)	UR	MR	MR	MR	MR	MR	LR	MR
Ahmed et al. [78] (2018)	UR	MR	MR	MR	MR	MR	LR	MR
Novoa et al. [52] (2020)	UR	LR	LR	MR	LR	LR	LR	MR
Cuenca et al. [15] (2021)	UR	LR	MR	MR	MR	LR	LR	MR
Willis et al. [51] (2020)	UR	MR	MR	MR	LR	LR	LR	MR
